# Trend of Prevalence of Atrial Fibrillation and use of Oral Anticoagulation Therapy in Patients With Atrial Fibrillation in South Korea (2002–2013)

**DOI:** 10.2188/jea.JE20160149

**Published:** 2018-02-05

**Authors:** Mi Kyoung Son, Nam-Kyoo Lim, Hyun-Young Park

**Affiliations:** Division of Cardiovascular and Rare Diseases, Center for Biomedical Science, Korea National Institute of Health, Chungcheongbuk-do, South Korea

**Keywords:** atrial fibrillation, prevalence, anticoagulation, comorbidity

## Abstract

**Background:**

This study examined the annual prevalence of atrial fibrillation (AF) and its associated comorbidities, as well as the prevalence of warfarin therapy in South Korean patients with AF.

**Methods:**

The National Health Insurance Service-National Sample Cohort database was searched for subjects aged ≥30 years diagnosed with AF from 2002–2013. The prevalence of AF was analyzed by sex and age, as was the current status of warfarin therapy in AF patients according to CHA_2_DS_2_-VASc score and comorbidities.

**Results:**

The age-standardized prevalence of AF in men and women was 0.15% and 0.14%, respectively, in 2002, increasing to 0.54% and 0.39%, respectively, in 2013. In 2013, the prevalence of AF in men and women aged 30–39 years was 0.08% and 0.03%, respectively, increasing to 2.35% and 1.71%, respectively, in those in aged ≥60 years. During 2002–2013, the prevalence of AF in men significantly increased among subjects aged ≥30 years and increased in women aged ≥60 years. The age-standardized prevalence of hypertension and diabetes mellitus among AF patients were markedly increased during 2002–2013. Of these AF patients, 86.1% had a CHA_2_DS_2_-VASc score of ≥2; however, only 39.1% of these were receiving warfarin.

**Conclusions:**

The age-standardized prevalence of AF increased 2.89-fold over the 12-year study period. The total number of patients with AF in South Korea has been drastically increasing, due to not only aging society but also increasing age-specific prevalence of AF, especially in middle-aged and elderly individuals. The rate of warfarin therapy increased slightly over the study period but remains low.

## INTRODUCTION

The incidence and prevalence of atrial fibrillation (AF) varies according to race and/or ethnicity and are expected to increase.^1^^–^^6^ In the United States, 2.3 million people are estimated to have AF, with the number expected to increase to 5.6 million by 2050.^1^ Assessment of Medicare beneficiaries reported that the incidence of AF over a 14-year period ranged from 27.3 to 28.3 per 1000 person-years, and that incidence rates were consistently higher in men than in women and among whites than in non-whites.^2^ In Asia, the prevalence of AF has been estimated to be approximately 1%.^1^^,^^3^ Estimates have suggested that, by 2050, 72 million individuals in Asia will be diagnosed with AF, more than double the combined numbers of patients in Europe and the United States, an increase attributable to the proportionally larger aged population in Asian countries.^4^^–^^6^ The previous studies for the trend in age-specific prevalence of AF were inconsistent. The studies in the United States, United Kingdom, and northern European populations showed that the prevalence of AF were significantly increased during study period among subjects aged ≥65 years,^2^^,^^7^^,^^8^ whereas the prevalence of AF in Japan were similar during study period among subjects aged ≥30 years both men and women.^9^

The Korean Heart Rhythm Society (KHRS) has recommended CHA_2_DS_2_-VASc instead of CHADS_2_ as a scoring system for the assessment of stroke risk.^10^ Oral anticoagulant (OAC) therapy with warfarin is associated with more frequent and costly monitoring and a higher risk of hemorrhage than other treatments, but it is significantly more effective at reducing stroke risk than antiplatelet agents, such as aspirin.^11^^–^^13^ According to the 2012 European Society of Cardiology (ESC) guidelines, AF patients with a CHA_2_DS_2_-VASc score ≥1 require ongoing thromboembolic prophylaxis with OAC therapy.^14^

The prevalence of AF is expected to increase as the Korean population ages, with AF likely to become a greater public health problem. Effective prevention and care of patients with AF require reliable determinations of its prevalence and incidence.^15^ However, to date, only a few epidemiologic studies have described the prevalence of AF in South Korea.^16^^,^^17^ The Korean Genome and Epidemiology Study (KoGES) found that the prevalence of AF was 1.0% in individuals aged 60–69 years,^16^ whereas another study reported a prevalence of 2.1% in subjects aged ≥65 years.^17^ As these studies enrolled populations aged 40–69 years^16^ or healthy individuals,^17^ they may have underestimated the prevalence of AF. Therefore, this study was designed to determine the annual prevalence of and comorbidities for AF during the years 2002–2013 and the current status of warfarin therapy according to stroke risk score (CHA_2_DS_2_-VASc score) in patients with AF using data from the database of the Korean National Health Insurance Service-National Sample Cohort (NHIS-NSC).

## METHODS

### Data source

We used data from the NHIS-NSC database, which consists of approximately one million medical insurance subscribers, who were selected using the stratified random sampling method with 1,476 strata by sex (2 strata), age (18 strata), and level of income (41 strata).^18^ This is equivalent to 2.2% of the entire South Korean population in 2002, and the subscribers were followed for 11 years until 2013, unless the subscriber was no longer considered eligible for health insurance due to death or emigration. During the follow-up period, the cohort was refreshed annually by adding a representative sample of newborns, sampled across 82 strata (2 for sex, combined with 41 for parents’ income levels) using the 2.2% sampling rate. The NHIS-NSC contains information on participants’ insurance eligibility and medical treatments. The insurance eligibility database also includes information on the participant’s identity and socioeconomic situation. The medical treatment database includes details of medical treatment, disease diagnoses codes, and prescriptions. The disease diagnoses codes were those of the Korean Classification of Diseases-6 (KCD-6), which is a modified version of the International Classification of Diseases-10 (ICD-10) for the Korean health care system. The study protocol was approved by the institutional review board of the Health Insurance Review and Assessment Service.

### Study population

The study population included subjects aged ≥30 years in each year. During 2002–2013, more than 570,000 subjects annually were eligible for analysis (eTable 1). The AF diagnosis code consists of the main code I48 (atrial fibrillation and atrial flutter), including two subcodes, I48.0 (atrial fibrillation) and I48.1 (atrial flutter). The physicians usually input I48 code (over 90%) for patients with atrial fibrillation or atrial flutter, instead of I48.0 or I48.1 codes. Patients were classified as AF prevalent if they had received at least two outpatient diagnosis of AF (KCD code I48, I48.0, I48.1) or at least one AF diagnosis during inpatient treatment, as previously reported.^2^^,^^19^ Comorbidities were retrospectively assessed in subjects subsequently diagnosed with AF by searching for the KCD-6 disease codes. CHA_2_DS_2_-VASc scores were divided into three categories: low (score 0), intermediate (score 1), and high (score ≥2).

### Statistical analysis

Continuous variables are expressed as mean (standard deviation [SD]) and compared using *t*-tests, and categorical variables are expressed as frequency (percentage) and compared using chi-square tests. The prevalence of AF was determined overall and by sex and age group during the 12-year period. The annual crude prevalence were calculated on December 31 of each year by dividing the number of patients alive with AF by the total number of subjects eligible for the NHIS-NSC during that year. The annual age-standardized prevalence of AF, both sex-combined and sex-specific, as well as the annual age-standardized prevalence of comorbidities according to the presence/absence of AF, were calculated using the direct method. The weights for the standardization were calculated using the proportions of the corresponding sex-combined and sex-specific age-groups in the 2015 Korean mid-year population that is the most recent available data (available from: http://kosis.kr/statHtml/statHtml.do?orgId=101&tblId=DT_1B040M5&conn_path=I3) (eTable 2). The Cochran-Armitage method was used to test for the linear trend in annual prevalence. All statistical tests were two-tailed, and *P*-values <0.05 were considered statistically significant. All statistical analyses were performed using SAS software (ver 9.4; SAS Institute, Cary, NC, USA).

## RESULTS

### Prevalence of AF

Table 1 shows the age-standardized and age-specific annual prevalence of AF during the 12-year study period. The prevalence of AF significantly increased over time (*P* for trend <0.001), being 0.15% (1,533 individuals) in 2002 and increasing to 0.47% (5213 individuals) in 2013. The prevalence of AF in men and women was 0.15% and 0.14%, respectively, in 2002, significantly increasing to 0.54% and 0.39%, respectively, in 2013 (*P* for trend <0.001). The prevalence of AF was higher in men than in women in all age groups. In 2013, the prevalence of AF in men and women aged 30–39 years was 0.08% and 0.03%, respectively, increasing to 4.80% and 3.41%, respectively, in those aged ≥80 years. The age-specific prevalence of AF in men significantly increased among subjects aged ≥30 years (*P* for trend >0.05) and was markedly increased in elderly subjects, increasing from 1.42% in 2002 to 4.80% in 2013 among subjects aged ≥80 years. By contrast, the prevalence did not change markedly in female patients aged <60 years (*P* for trend >0.05), being 0.03%, 0.08%, and 0.26% in 2002 and 0.03%, 0.08%, and 0.32% in 2013, among subjects aged 30–39 years, 40–49 years, and 50–59 years, respectively. The age-specific prevalence of AF markedly increased in elderly female subjects, increasing from 0.93% in 2002 to 3.41% in 2013.

**Table 1.** Age-standardized and age-specific prevalence of AF stratified by age group and sex during 2002–2013

Age group	Year												*P* for trend

	2002 (*n* = 575,969)	2003 (*n* = 585,795)	2004 (*n* = 595,236)	2005 (*n* = 604,474)	2006 (*n* = 605,118)	2007 (*n* = 623,703)	2008 (*n* = 618,178)	2009 (*n* = 626,145)	2010 (*n* = 637,928)	2011 (*n* = 649,342)	2012 (*n* = 660,209)	2013 (*n* = 670,040)	
Total^a^	1,533 (0.15)	2,070 (0.20)	2,409 (0.23)	2,740 (0.26)	2,816 (0.26)	3,086 (0.28)	3,361 (0.31)	3,631 (0.33)	3,844 (0.35)	4,446 (0.40)	4,822 (0.43)	5,213 (0.47)	<0.001
Male^a^	802 (0.15)	1,087 (0.21)	1,286 (0.25)	1,497 (0.28)	1,551 (0.29)	1,704 (0.31)	1,916 (0.35)	1,999 (0.37)	2,130 (0.39)	2,469 (0.46)	2,686 (0.50)	2,943 (0.54)	<0.001
30–39 years	31 (0.03)	45 (0.05)	53 (0.06)	71 (0.08)	61 (0.07)	62 (0.07)	57 (0.07)	48 (0.06)	41 (0.05)	50 (0.06)	56 (0.07)	62 (0.08)	0.004
40–49 years	90 (0.11)	132 (0.15)	155 (0.17)	160 (0.18)	155 (0.17)	150 (0.17)	177 (0.20)	170 (0.19)	164 (0.18)	186 (0.21)	205 (0.23)	194 (0.22)	<0.001
50–59 years	169 (0.35)	215 (0.44)	253 (0.49)	305 (0.55)	315 (0.55)	323 (0.53)	347 (0.55)	366 (0.56)	383 (0.55)	485 (0.65)	513 (0.66)	563 (0.70)	<0.001
60–69 years	293 (0.85)	386 (1.08)	439 (1.21)	482 (1.33)	493 (1.35)	513 (1.34)	580 (1.50)	614 (1.56)	651 (1.61)	685 (1.69)	681 (1.63)	752 (1.74)	<0.001
70–79 years	169 (1.32)	233 (1.66)	291 (1.92)	364 (2.22)	403 (2.232)	499 (2.64)	553 (2.81)	584 (2.78)	644 (2.89)	778 (3.27)	909 (3.52)	1,006 (3.73)	<0.001
≥80 years	50 (1.42)	76 (1.76)	95 (2.12)	115 (2.45)	124 (2.54)	157 (3.02)	202 (3.78)	217 (3.78)	247 (3.97)	285 (4.32)	322 (4.53)	366 (4.80)	<0.001
Female^a^	731 (0.14)	983 (0.19)	1,123 (0.21)	1,243 (0.22)	1,265 (0.23)	1,382 (0.24)	1,445 (0.26)	1,632 (0.30)	1,714 (0.31)	1,977 (0.34)	2,136 (0.37)	2,270 (0.39)	<0.001
30–39 years	24 (0.03)	29 (0.03)	35 (0.04)	35 (0.04)	26 (0.03)	32 (0.04)	30 (0.04)	33 (0.04)	31 (0.04)	27 (0.03)	26 (0.03)	24 (0.03)	0.591
40–49 years	68 (0.08)	78 (0.09)	72 (0.08)	86 (0.10)	71 (0.08)	71 (0.08)	82 (0.09)	86 (0.10)	75 (0.09)	77 (0.09)	76 (0.09)	73 (0.08)	0.994
50–59 years	125 (0.26)	168 (0.34)	168 (0.33)	176 (0.32)	199 (0.35)	189 (0.31)	170 (0.27)	206 (0.32)	230 (0.33)	232 (0.31)	243 (0.32)	255 (0.32)	0.574
60–69 years	246 (0.60)	315 (0.75)	353 (0.83)	345 (0.82)	344 (0.83)	385 (0.89)	421 (0.98)	420 (0.97)	426 (0.97)	439 (1.00)	450 (1.01)	476 (1.03)	<0.001
70–79 years	187 (0.84)	264 (1.12)	328 (1.31)	413 (1.57)	408 (1.48)	461 (1.59)	480 (1.65)	564 (1.86)	597 (1.89)	727 (2.19)	796 (2.27)	832 (2.30)	<0.001
≥80 years	81 (0.93)	129 (1.25)	167 (1.57)	188 (1.68)	217 (1.88)	244 (1.96)	262 (2.05)	323 (2.36)	355 (2.42)	475 (3.04)	545 (3.24)	610 (3.41)	<0.001

Data reported as number of patients with AF (%).

^a^Data reported as number of patients with AF (age-standardized prevalence, %).

*P*-value obtained by Cochran-Armitage trend test.

### Comorbidities for AF

Figure 1-1 and Figure 1-2 show the age-standardized prevalence and crude number, respectively, of patients with comorbidities for AF by sex during 2002–2013. The detailed results for the crude and age-standardized prevalence of comorbidities for patients with and without AF by sex are shown in eTable 3. Both hypertension and diabetes were markedly increased during that period. The age-standardized prevalence of hypertension among men and women with AF significantly increased from 71.2% to 79.4% and from 74.2% to 83.0%, respectively, while the age-standardized prevalence of hypertension among men and women without AF significantly increased from 6.6% to 18.5% and from 8.7% to 17.8%, respectively. The age-standardized prevalence of diabetes mellitus among those with AF significantly increased from 25.8% to 35.8% in men and from 25.0% to 38.4% in women, respectively, while the corresponding prevalence among those without AF significantly increased from 4.7% to 9.8% in men and from 4.7% to 8.8% in women, respectively. During 2002–2013, the number of men with hypertension and diabetes mellitus who had AF increased by 1851 and 940 individuals, respectively, and corresponding numbers of women increased by 1375 and 722 individuals, respectively. The age-standardized prevalence of heart failure, ischemic heart disease (IHD), and valvular heart disease (VHD) significantly decreased by 11.7%, 10.8%, and 6.2%, respectively, in men with AF and by 11.1%, 8.2%, and 6.4%, respectively, in women with AF. However, the number of men with heart failure, IHD, VHD, and cardiomyopathy increased by 422, 758, 146, and 113, respectively, and the number of women with these comorbidities increased by 426, 475, 207, and 43, respectively, during 2002–2013. After adjusted for age in 2013, 25.6% of patients with AF had heart failure, 80.9% had hypertension, 36.9% had diabetes mellitus, 34.9% had IHD, 12.7% had VHD, and 4.8% had cardiomyopathy. Of the patients without AF in 2013, 1.0% had heart failure, 18.2% had hypertension, 9.3% had diabetes mellitus, 3.4% had IHD, 0.2% had VHD, and 0.1% had cardiomyopathy.

**Figure 1-1.**
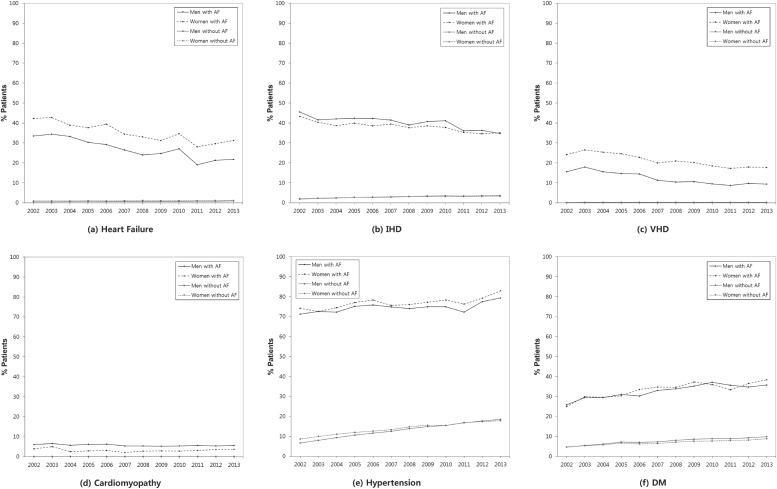
Age-standardized prevalence of comorbidities in AF patients by sex during 2002–2013

**Figure 1-2.**
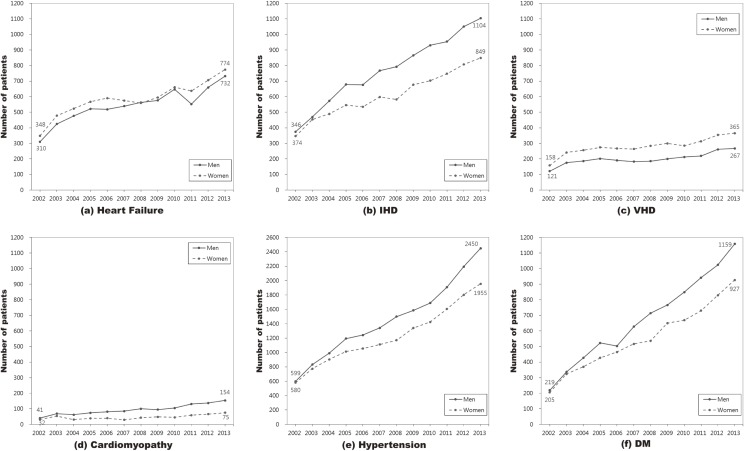
Number of patients with comorbidities in AF patients by sex during 2002–2013

### Distribution of stroke risk score and OAC therapy in patients with AF

**Figure 2.**
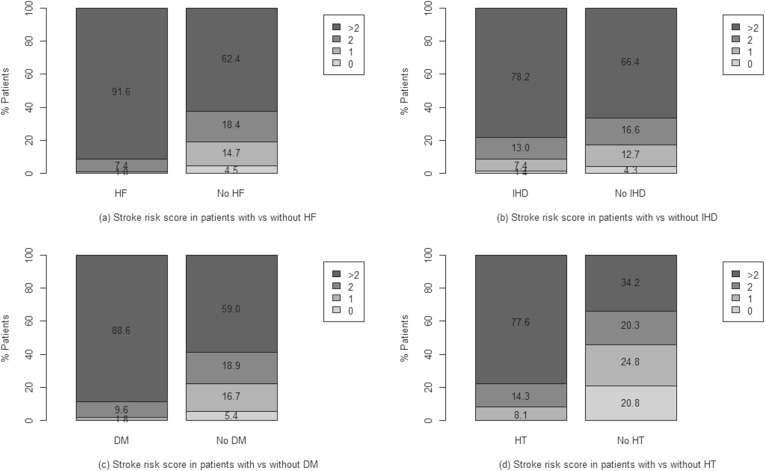
Age-standardized rates of CHA_2_DS_2_-VASc scores in 2013 in atrial fibrillation patients with and without (a) heart failure (HF), (b) ischemic heart disease (IHD), (c) diabetes mellitus (DM), and (d) hypertension (HT)

The proportion of AF patients with CHADS_2_ and CHA_2_DS_2_-VASc scores ≥2 increased from 62.1% and 80.7% in 2002 to 71.2% and 86.1% in 2013, respectively, and the rate of warfarin treatment of patients with AF increased from 27.5% in 2002 to 37.0% in 2013 (eTable 4). Table 2 shows the warfarin use of patients with AF in 2013. Of the AF patients with CHA_2_DS_2_-VASc scores ≥2 in 2013, only 39.1% were receiving warfarin therapy. Fewer than 30% of patients with CHA_2_DS_2_-VASc scores ≤1 received warfarin therapy. The distributions of CHA_2_D_2_-VASc score according to the presence of comorbidities are shown in Figure 2. Of the AF patients without heart failure, IHD, diabetes mellitus, and hypertension, 80.8%, 83.0%, 77.9%, and 54.5%, respectively, had CHA_2_D_2_-VASc scores ≥2. Of the AF patients with heart failure, IHD, diabetes mellitus, and hypertension, 99.0%, 91.2%, 98.2%, and 91.9%, respectively, had CHA_2_D_2_-VASc scores ≥2. However, warfarin treatment was more common in AF patients with VHD (68.0%) than AF patients with other comorbidities, such as heart failure (41.7%), hypertension (38.2%), IHD (34.8%), and diabetes mellitus (39.1%). Warfarin therapy differed significantly among groups stratified by age and CHA_2_DS_2_-VASc score, as well as by the presence of heart failure, hypertension, diabetes mellitus, IHD, VHD, and cardiomyopathy. The warfarin treatment rates in patients aged <60 years and ≥60 years were 32.4% and 38.3%, respectively, with no difference by gender.

**Table 2.** The OAC use according to the characteristics of patients with AF in 2013

Characteristics	Oral Anticoagulant Therapy		*P*-value^a^

	Yes	No	
Total	1,929 (37.0)	3,284 (63.0)	
Age			<0.001
30–39 years	18 (20.9)	68 (79.1)	
40–49 years	96 (36.0)	171 (64.0)	
50–59 years	265 (32.4)	553 (67.6)	
60–69 years	473 (38.5)	755 (61.5)	
70–79 years	783 (42.6)	1,055 (57.4)	
≥80 years	294 (30.1)	682 (69.9)	
Sex			0.417
Male	1,075 (36.5)	1,868 (63.5)	
Female	854 (37.6)	1,416 (62.4)	
CHA_2_DS_2_-VASc			<0.001
Low (score 0)	31 (18.5)	137 (81.5)	
Intermediate (score 1)	145 (25.9)	414 (74.1)	
High (score ≥2)	1,753 (39.1)	2,733 (60.9)	
Mean (SD)	3.94 (1.83)	3.52 (2.00)	<0.001
Heart failure	628 (41.7)	878 (58.3)	<0.001
Hypertension	1,683 (38.2)	2,722 (61.8)	<0.001
Diabetes mellitus	815 (39.1)	1,271 (60.9)	0.012
IHD	680 (34.8)	1,273 (65.2)	0.011
VHD	430 (68.0)	202 (32.0)	<0.001
Cardiomyopathy	120 (52.4)	109 (47.6)	<0.001

AF, atrial fibrillation; IHD, ischemic heart disease; VHD, valvular heart disease.

Data reported as *n* (%).

^a^by chi-square test for categorical variables and *t*-test for continuous variables.

## DISCUSSION

To our knowledge, this is the first report on the annual prevalence of AF and warfarin therapy in South Korea using a nationwide population-based cohort study. We found that the prevalence of AF increased over the last decade, especially in older patients, and was higher in men than in women in all age groups. Particularly, the prevalence of AF significantly increased in men aged ≥30 years and in women aged ≥60 years. However, the rate of warfarin therapy in the high risk group in 2013 was much lower than that in other countries.^7^^,^^20^^,^^21^

NHIS-NSC is a cohort based on nationwide health insurance data, so it is both representative of the population and overcomes the limitations of cross-sectional data. Information pertaining to patients and their medical records is available in the South Korean NHIS database,^21^ which was used previously to conduct an epidemiological study.^22^ Our assessment of the NHIS-NSC database showed that the prevalence of AF increased progressively with age and was higher among men than women in all age groups, and overall prevalence of AF increased every year (from 0.27% in 2002 to 0.78% in 2013), particularly in men aged ≥30 years and women aged ≥60 years, findings which were inconsistent with previous studies^8^^,^^9^ and the first report. Prevalence of AF in Europe and the United States was 3–4% in subjects aged 65–69 years and 16–18% in subjects aged ≥85 years, rates which were higher than our results.^2^^,^^23^ In Japan, the overall prevalence of AF was 0.56%, and the prevalence of AF was 4.4% in men and 2.2% in women aged ≥80 years.^24^ In China, the prevalence of AF was 4.8% in men and 2.6% in women aged 75–79 years.^25^ Assessment of Medicare beneficiaries in the United States showed that the prevalence of AF increased during 1993–2007 among subjects aged >65 years.^2^ Similarly, Doctor’s Independent Network database (DIN-LINK) shows a steady rise in the trend in prevalence of AF during 1994–2003 in both men and women aged ≥65 years, and the increase in men was higher than in women in all age groups during that period.^7^ On the other hand, in Japan, trends in prevalence of AF among men and women aged ≥30 years were similar during 1980–2000.^9^ In other study in a northern European population during 1998–2008, the prevalence of AF was similar in subjects aged <65 years but significantly increased in subjects aged ≥65 years.^8^

A 1-year prospective cohort study in England and Wales showed that the risk factors for AF were IHD, heart failure, and hypertension.^26^ The risks of AF in men and women were increased 1.5- and 1.4-fold, respectively, by hypertension and 4.5- and 5.9-fold, respectively, by heart failure.^27^ VHD and cardiomyopathy were found to be important risk factors for AF,^28^ with VHD associated with 1.8- and 3.4-fold risks for AF in men and women, respectively.^15^ To assess the reason for the increasing trend in annual age-specific prevalence of AF, the present study also revealed comorbidities (heart failure, hypertension, IHD, diabetes mellitus, VHD, and cardiomyopathy) among AF patients during the 12-year period. Hypertension was more prevalent than other comorbidities during 2002–2013, ranging from 76.9% to 84.5%. During that period, the percentages of subjects with hypertension and diabetes mellitus increased, while the percentages with heart failure, IHD, and VHD decreased. Among AF patients, the number of patients with hypertension, diabetes mellitus, and IHD sharply increased during study period. In 2013, the prevalences of comorbidities in patients with AF were more frequent in women than in men, except for cardiomyopathy.

The reasons for the increasing trend in prevalence of AF are multifactorial. First, the population in South Korean is undergoing rapid aging; indeed, South Korea has the fastest aging population among Organization for Economic Cooperation and Development (OECD) countries. Second, increased use of routine electrocardiograms may have enhanced the detection of AF. Third, the increased prevalence of AF comorbidities, such as hypertension, diabetes mellitus, and IHD, as well as the higher BMI among AF patients, may have contributed to the increase in the prevalence of AF. Previous studies reported that the incidence of AF is associated with hypertension, diabetes mellitus, IHD, and BMI in South Korea,^29^^,^^30^ and that higher BMI was significantly increased in men aged ≥20 years and in women aged ≥60 years during 1998–2007.^31^ Also, we found that the prevalence of hypertension and diabetes mellitus among AF patients aged ≥60 years significantly increased during 2002–2013 (eTable 3), and the increase in the number of AF patients with hypertension, diabetes mellitus, and IHD was higher for men than for women in all age group (eTable 5). It seems likely that the prevalence of AF will continue to increase, potentially making AF an important public health problem in the future.

OAC with warfarin, a synthetic derivative of coumarin, is reported to reduce the risk of ischemic stroke by 64–70%,^14^^,^^32^ an additional 30–50% compared with aspirin.^33^ The rate of OAC use in patients with AF varies across countries. The use of OAC in Europe and North America were higher than in other regions (about 50% in the USA, 67% in Europe, 75% in Japan, 2.5% in China, 49% in the Gulf states, 58% in Brazil, and 30% in Cameroon in patients with CHADS_2_ or CHA_2_DS_2_-VASc score ≥2), but OAC was still underused, owing to perceived risks and concerns.^21^ In the present study, the rate of warfarin therapy in patients with AF increased from 21.4% in 2002 to 33.1% in 2013, but this rate remained low. We found that, in 2013, only 35.5% of patients with a CHA_2_DS_2_-VASc score ≥2 received warfarin therapy. Rates of warfarin therapy were very low (<40% each) in patients with heart failure or IHD. We found that 32.0% of AF patients were receiving warfarin therapy in 2012; of these, 73.8% were treated for ≥1 year, whereas 2.1% were treated for <1 month (data not shown). Recently, non-vitamin K antagonist oral anticoagulant or direct oral anticoagulant (NOAC or DOAC), such as rivaroxaban, apixaban, edoxaban, and dabigatran, have been approved as OACs.^34^^–^^37^ The ESC and National Institute for Health and Care Excellence (NICE) guidelines allow greater opportunities for stroke prevention, especially with NOACs, based on CHA_2_DS_2_-VASc score.^14^^,^^38^ As NOACs have been reimbursed by the Korean National Health Insurance since 2015, their use is expected to increase. We guess that many patients who newly developed AF in recent years did not have underlying disease and had relatively low CHA_2_DS_2_-Vasc scores. To administer NOAC instead of warfarin for AF patients with relatively low CHA_2_DS_2_-VASc scores is likely to be an important issue in South Korea in the near future.

This study had several limitations. First, as patients with AF were only identified according to KCD-6 codes, this group included patients with atrial flutter, given correlation between the two dysrhythmias. We also could not distinguish between patients with persistent and paroxysmal AF, thus some patients with AF may only have had a single episode of paroxysmal AF. However, individuals with an index AF event have high rates of recurrence and conversion to persistent AF.^39^ Moreover, paroxysmal and persistent AF are associated with a similar risk for stroke.^40^ Second, the possibility of misclassification for diagnoses might exist in this study because the diagnoses for AF or comorbidities were defined based on KCD-6 codes, but we were unable to validate the AF diagnosis using electrocardiograms or other medical data. Instead, we used a stricter definition of AF. As previously reported, patients who had only received one outpatient diagnosis of AF were not considered definitely to be AF patients,^2^^,^^19^ in order to minimize the impact of rule-out diagnoses and to improve the specificity of our definition of AF. Moreover, a recent validation study of the Korean NHIS database showed that the accuracy of diagnosis codes tended to be higher for claims from hospital admissions than from office visits, and for claims for severe than for mild conditions.^41^^,^^42^ Third, we could not evaluate the use of NOAC therapy because these agents were not included in our prescription drug code.

Nevertheless, our study had several strengths, including the sample size, which was larger than in previous studies. Furthermore, to our knowledge, our study is the first to investigate the annual prevalence of AF, its comorbidities, and patterns of anticoagulation therapy based on stroke risk (CHA_2_DS_2_-VASc) score using a nationwide database in South Korea.

In conclusion, this nationwide survey on the prevalence of AF using medical claim data from the NHIS-NSC has shown that the prevalence of AF increased during the 12-year study period, and that the increase in number of patients with AF was attributable to both aging society and increasing age-specific prevalence of AF, especially in middle-aged and elderly individuals. Also, considerable increases in prevalence of hypertension and diabetes mellitus might contribute to a marked increase in the number of patients with AF in South Korea. Reducing the prevalence rates of hypertension and diabetes is an urgent task in order to reduce rates not only of coronary artery disease and stroke but also of AF. Also, as the South Korean population continues to age and AF comorbidities change, it seems likely that the prevalence of AF will increase, potentially giving rise to a major public health problem in the years ahead. The rate of warfarin therapy among AF patients with CHA_2_DS_2_-VASc scores ≥2 remained low in 2013, indicating a need to increase warfarin treatment of AF patients. The results of this study could provide a foundation for the evaluation of preventative measures and treatments of AF in South Korea. Moreover, mass screenings using electrocardiography recordings are needed to determine whether systematic screening for untreated AF and initiation of OAC therapy can reduce the risk of ischemic stroke in a cost-effective manner.

## APPENDIX A. SUPPLEMENTARY DATA

eTable 1. Characteristics of study population, 2002–2013

eTable 2. Weights for age-standardized prevalence, 2015 Korea midyear distribution

eTable 3-1. Crude and age-standardized prevalence of comorbidities in overall by with and without AF during 2002–2013

eTable 3-2. Crude and age-standardized prevalence of comorbidities in men by with and without AF during 2002–2013

eTable 3-3. Crude and age-standardized prevalence of comorbidities in women by with and without AF during 2002–2013

eTable 4. CHADS_2_ and CHA_2_DS_2_-VASc score and oral anticoagulant therapy among AF patients during 2002–2013

eTable 5. Age-specific prevalence of hypertension, DM and IHD by sex and age group among AF patients during 2002–2013
