# Supplementary material for: Trend of Prevalence of Atrial Fibrillation and use of Oral Anticoagulation Therapy in Patients With Atrial Fibrillation in South Korea (2002–2013)
Source: J Epidemiol. 2018 Feb 5;28(2):81–7. doi: 10.2188/jea.JE20160149 (PMC5792231; doi:10.2188/jea.JE20160149)
Supplement: Supplementary file 1 [file je-28-081-s001.pdf]

eTable 1. Characteristics of study population, 2002–2013

Characteristics	2002 (n=575,969)	2003 (n=585,795)	2004 (n=595,236)	2005 (n=604,474)	2006 (n=605,118)	2007 (n=623,703)	2008 (n=618,178)	2009 (n=626,145)	2010 (n=637,928)	2011 (n=649,342)	2012 (n=660,209)	2013 (n=670,040)
**Age group**												
30–39 years	187,717 (32.59)	185,841 (31.72)	184,670 (31.02)	182,248 (30.15)	177,226 (29.29)	177,460 (28.45)	169,060 (27.35)	165,369 (26.41)	162,987 (25.55)	160,897 (24.78)	158,860 (24.06)	155,953 (23.28)
40–49 years	168,327 (29.23)	170,973 (29.19)	173,700 (29.18)	174,979 (28.95)	173,915 (28.74)	177,551 (28.47)	176,281 (28.52)	176,397 (28.17)	175,856 (27.57)	175,264 (26.99)	175,171 (26.53)	176,668 (26.37)
50–59 years	97,116 (16.86)	98,979 (16.90)	103,045 (17.31)	110,350 (18.26)	114,722 (18.96)	121,765 (19.52)	124,410 (20.13)	131,086 (20.94)	139,950 (21.94)	149,533 (23.03)	154,715 (23.43)	159,483 (23.80)
60–69 years	75,522 (13.11)	77,791 (13.28)	78,573 (13.20)	78,236 (12.94)	77,926 (12.88)	81,402 (13.05)	81,543 (13.19)	82,541 (13.18)	84,360 (13.22)	84,453 (13.01)	86,658 (13.13)	89,336 (13.33)
70–79 years	35,064 (6.09)	37,609 (6.42)	40,132 (6.74)	42,755 (7.07)	44,884 (7.42)	47,896 (7.68)	48,774 (7.89)	51,351 (8.20)	53,869 (8.44)	56,996 (8.78)	60,886 (9.22)	63,076 (9.41)
≥80 years	12,223 (2.12)	14,602 (2.49)	15,116 (2.54)	15,906 (2.63)	16,445 (2.72)	17,629 (2.83)	18,110 (2.93)	19,401 (3.10)	20,906 (3.28)	22,199 (3.42)	23,919 (3.62)	25,524 (3.81)
**Sex**												
Male	280,535 (48.71)	285,560 (48.75)	290,034 (48.73)	294,556 (48.73)	294,944 (47.74)	303,892 (48.72)	302,199 (48.89)	306,011 (48.87)	311,793 (48.88)	317,315 (48.87)	322,616 (48.87)	327,515 (48.88)
Female	295,434 (51.29)	300,235 (51.25)	305,202 (51.27)	309,918 (51.27)	310,174 (51.26)	319,811 (51.28)	315,979 (51.11)	320,134 (51.13)	326,135 (51.12)	332,027 (51.13)	337,533 (51.13)	342,525 (51.12)
**AF**	1,533 (0.27)	2,070 (0.35)	2,409 (0.40)	2,740 (0.45)	2,816 (0.47)	3,086 (0.49)	3,361 (0.54)	3,631 (0.58)	3,844 (0.60)	4,446 (0.68)	4,822 (0.73)	5,213 (0.78)
**HF**	7,539 (1.31)	8,460 (1.44)	9,026 (1.52)	9,749 (1.61)	9,438 (1.56)	10,135 (1.62)	10,463 (1.69)	10,397 (1.66)	10,504 (1.65)	10,860 (1.67)	11,357 (1.72)	11,948 (1.78)
**IHD**	16,961 (2.94)	20,450 (3.49)	22,737 (3.82)	25,530 (4.22)	25,373 (4.19)	27,766 (4.45)	29,876 (4.83)	31,665 (5.06)	31,971 (5.01)	32,354 (4.98)	33,091 (5.01)	34,305 (5.12)
**VHD**	1,280 (0.22)	1,547 (0.26)	1,603 (0.27)	1,683 (0.28)	1,678 (0.28)	1,724 (0.28)	1,842 (0.30)	1,877 (0.30)	1,878 (0.29)	2,228 (0.34)	2,372 (0.36)	2,338 (0.35)
**Cardiomyopathy**	498 (0.09)	597 (0.10)	546 (0.09)	601 (0.10)	576 (0.10)	648 (0.10)	693 (0.11)	724 (0.12)	738 (0.12)	875 (0.13)	1,028 (0.16)	1,042 (0.16)
**Hypertension**	68,660 (11.92)	81,352 (13.89)	92,454 (15.53)	102,992 (17.04)	109,004 (18.01)	118,084 (18.93)	129,052 (20.88)	136,693 (21.83)	136,998 (21.48)	152,239 (23.45)	159,376 (24.14)	164,901 (24.61)
**DM**	38,005 (6.60)	45,068 (7.69)	49,992 (8.40)	58,061 (9.61)	56,452 (9.33)	60,223 (9.66)	65,103 (10.53)	69,363 (11.08)	70,716 (11.09)	72,428 (11.15)	76,410 (11.57)	81,284 (12.13)

Data reported as n(%)

AF, atrial fibrillation; IHD, ischemic heart disease; HF, heart failure; DM, diabetes mellitus; VHD, valvular heart disease.

Table 2. Weights for age-standardized prevalence, 2015 Korea midyear distribution

Age group	Overall	Men	Women
**30–39 years**	0.227	0.237	0.217
**40–49 years**	0.259	0.269	0.250
**50–59 years**	0.241	0.248	0.234
**60–69 years**	0.142	0.142	0.143
**70–79 years**	0.092	0.081	0.104
**≥ 80 years**	0.039	0.024	0.053

eTable 3-1. Crude and age-standardized prevalence of comorbidities in overall by with and without AF during 2002–2013

Comorbidities	Year												*P* for trend
	2002	2003	2004	2005	2006	2007	2008	2009	2010	2011	2012	2013	
**HF**													
With AF	42.9 / 37.6	43.6 / 38.2	41.5 / 35.7	39.7 / 33.4	39.4 / 33.5	36.1 / 29.9	33.4 / 27.7	32.3 / 27.5	34.0 / 30.3	26.7 / 22.7	28.3 / 24.7	28.9 / 25.6	<0.001
Without AF	1.2 / 0.7	1.3 / 0.7	1.4 / 0.8	1.4 / 0.8	1.4 / 0.8	1.5 / 0.8	1.5 / 0.9	1.5 / 0.9	1.5 / 0.9	1.5 / 0.9	1.5 / 0.9	1.6 / 1.0	<0.001
**IHD**													
With AF	47.0 / 44.7	44.5 / 41.1	44.1 / 40.6	44.7 / 41.4	43.0 / 40.7	44.2 / 40.6	40.9 / 38.4	42.5 / 39.8	42.5 / 39.7	38.3 / 35.7	38.5 / 35.6	37.5 / 34.9	<0.001
Without AF	2.8 / 1.9	3.3 / 2.2	3.7 / 2.4	4.0 / 2.7	4.0 / 2.7	4.3 / 2.9	4.6 / 3.2	4.8 / 3.3	4.8 / 3.3	4.8 / 3.3	4.8 / 3.3	4.9 / 3.4	<0.001
**VHD**													
With AF	18.2 / 19.5	20.1 / 21.8	18.4 / 19.8	17.3 / 18.9	16.3 / 17.9	14.4 / 15.0	14.0 / 14.8	13.7 / 14.7	12.9 / 13.3	12.0 / 12.3	12.8 / 13.1	12.1 / 12.7	<0.001
Without AF	0.2 / 0.1	0.2 / 0.1	0.2 / 0.1	0.2 / 0.1	0.2 / 0.1	0.2 / 0.1	0.2 / 0.2	0.2 / 0.2	0.2 / 0.2	0.3 / 0.2	0.3 / 0.2	0.3 / 0.2	<0.001
**Cardiomyopathy**													
With AF	4.8 / 5.0	5.9 / 5.8	3.9 / 4.3	4.1 / 4.7	4.3 / 4.9	3.7 / 3.9	4.3 / 4.3	3.9 / 4.2	3.9 / 4.3	4.3 / 4.6	4.2 / 4.6	4.4 / 4.8	0.111
Without AF	0.1 / 0.0	0.1 / 0.1	0.1 / 0.1	0.1 / 0.1	0.1 / 0.1	0.1 / 0.1	0.1 / 0.1	0.1 / 0.1	0.1 / 0.1	0.1 / 0.1	0.1 / 0.1	0.1 / 0.1	<0.001
**Hypertension**													
With AF	76.9 / 72.6	77.6 / 72.6	78.7 / 73.2	80.6 / 76.0	81.6 / 76.9	79.5 / 75.1	79.4 / 74.9	80.6 / 75.9	81.0 / 76.4	79.0 / 73.9	82.9 / 78.2	84.5 / 80.9	<0.001
Without AF	11.7 / 7.6	13.7 / 8.9	15.3 / 10.1	16.7 / 11.2	17.7 / 12.1	18.6 / 12.9	20.6 / 14.3	21.5 / 15.3.	21.1 / 15.4	23.1 / 16.9	23.7 / 17.6	24.1 / 18.2	<0.001
**DM**													
With AF	27.7 / 25.4	31.9 / 29.7	33.1 / 29.5	34.7 / 30.8	34.4 / 31.6	37.1 / 33.8	37.2 / 34.2	39.0 / 36.1	39.5 / 36.6	37.6 / 34.7	38.4 / 35.5	40.0 / 36.9	<0.001
Without AF	6.5 / 4.7	7.6 / 5.5	8.3 / 6.0	9.5 / 6.9	9.2 / 6.7	9.5 / 6.9	10.4 / 7.6	10.9 / 8.1	10.9 / 8.3	11.0 / 8.3	11.4 / 8.8	11.9 / 9.3	<0.001

Data reported as crude prevalence, % / age-standardized prevalence, %
DM, diabetes mellitus; HF, heart failure; IHD, ischemic heart disease; VHD, valvular heart disease.
P-value obtained by Cochran-Armitage trend test

eTable 3-2. Crude and age-standardized prevalence of comorbidities in men by with and without AF during 2002–2013

Comorbidities	Year												*P* for trend
	2002	2003	2004	2005	2006	2007	2008	2009	2010	2011	2012	2013	
**HF**													
With AF	38.7 / 33.5	39.0 / 34.4	37.0 / 33.2	34.8 / 30.3	33.4 / 29.2	31.6 / 26.5	29.4 / 24.0	28.8 / 24.7	30.4 / 27.0	22.4 / 19.0	24.5 / 21.3	24.9 / 21.8	<0.001
Without AF	0.8 / 0.5	0.9 / 0.5	1.0 / 0.6	1.1 / 0.6	1.1 / 0.6	1.1 / 0.7	1.2 / 0.7	1.2 / 0.7	1.2 / 0.7	1.2 / 0.8	1.3 / 0.8	1.4 / 0.9	<0.001
**IHD**													
With AF	46.6 / 45.5	43.1 / 41.6	44.6 / 42.0	45.3 / 42.4	43.6 / 42.3	45.0 / 41.5	41.3 / 39.0	43.3 / 40.7	43.7 / 41.1	38.6 / 36.1	39.1 / 36.3	37.5 / 34.7	<0.001
Without AF	2.7 / 1.8	3.2 / 2.2	3.6 / 2.4	4.0 / 2.7	4.1 / 2.8	4.3 / 2.9	4.7 / 3.2	4.9 / 3.4	5.0 / 3.5	4.9 / 3.4	4.9 / 3.5	5.0 / 3.6	<0.001
**VHD**													
With AF	15.1 / 15.5	16.1 / 17.9	14.5 / 15.5	13.4 / 14.7	12.3 / 14.4	10.7 / 11.3	9.7 / 10.4	10.0 / 10.6	10.0 / 9.5	8.9 / 8.7	9.7 / 9.7	9.1 / 9.3	<0.001
Without AF	0.1 / 0.1	0.2 / 0.1	0.2 / 0.1	0.2 / 0.1	0.2 / 0.1	0.2 / 0.1	0.2 / 0.1	0.2 / 0.1	0.2 / 0.1	0.2 / 0.1	0.2 / 0.1	0.2 / 0.1	<0.001
**Cardiomyopathy**													
With AF	5.1 / 6.0	6.3 / 6.6	4.8 / 5.7	4.9 / 6.1	5.2 / 6.2	5.0 / 5.3	5.2 / 5.4	4.8 / 5.2	4.9 / 5.3	5.3 / 5.6	5.1 / 5.3	5.2 / 5.6	0.377
Without AF	0.1 / 0.0	0.1 / 0.1	0.1 / 0.1	0.1 / 0.1	0.1 / 0.1	0.1 / 0.1	0.1 / 0.1	0.1 / 0.1	0.1 / 0.1	0.1 / 0.1	0.1 / 0.1	0.1 / 0.1	<0.001
**Hypertension**													
With AF	74.7 / 71.2	76.5 / 72.6	77.1 / 72.2	79.8 / 75.2	80.1 / 75.8	78.8 / 74.8	78.2 / 74.0	79.3 / 75.0	79.3 / 74.9	77.3 / 72.2	81.7 / 77.5	83.2 / 79.4	<0.001
Without AF	10.1 / 6.6	12.1 / 8.0	13.8 / 9.3	15.4 / 10.6	16.6 / 11.6	17.6 / 12.5	19.2 / 13.9	20.3 / 14.9	20.5 / 15.4	22.2 / 16.8	23.0 / 17.7	23.7 / 18.5	<0.001
**DM**													
With AF	27.3 / 25.8	31.0 / 29.5	33.2 / 29.5	34.9 / 31.0	32.4 / 30.2	36.9 / 33.1	37.3 / 33.9	38.3 / 35.2	39.9 / 37.1	38.2 / 35.6	38.1 / 34.7	39.4 / 35.8	<0.001
Without AF	6.5 / 4.7	7.7 / 5.5	8.4 / 6.1	9.7 / 7.1	9.5 / 7.0	9.9 / 7.3	10.8 / 8.0	11.3 / 8.6	11.5 / 8.8	11.3 / 8.8	11.8 / 9.3	12.3 / 9.8	<0.001

Data reported as crude prevalence, % / age-standardized prevalence, %
DM, diabetes mellitus; HF, heart failure; IHD, ischemic heart disease; VHD, valvular heart disease.
P-value obtained by Cochran-Armitage trend test

eTable 3-3. Crude and age-standardized prevalence of comorbidities in women by with and without AF during 2002–2013

Comorbidities	Year												*P* for trend
	2002	2003	2004	2005	2006	2007	2008	2009	2010	2011	2012	2013	
**HF**													
With AF	47.6 / 42.3	48.6 / 42.7	46.6 / 38.9	45.6 / 37.7	46.6 / 39.5	41.6 / 34.4	38.7 / 33.0	36.5 / 31.2	38.6 / 34.6	32.2 / 28.1	33.1 / 29.6	34.1 / 31.2	<0.001
Without AF	1.5 / 0.9	1.6 / 0.9	1.7 / 0.9	1.7 / 1.0	1.7 / 0.9	1.8 / 1.0	1.9 / 1.1	1.8 / 1.0	1.7 / 1.0	1.8 / 1.0	1.8 / 1.1	1.8 / 1.1	<0.001
**IHD**													
With AF	47.3 / 43.3	46.1 / 40.4	43.5 / 38.7	43.9 / 39.9	42.2 / 38.6	43.3 / 39.4	40.3 / 37.6	41.5 / 38.5	41.0 / 37.8	37.8 / 35.3	37.4 / 34.6	41.0 / 35.1	<0.001
Without AF	3.0 / 2.0	3.5 / 2.3	3.7 / 2.5	4.1 / 2.7	4.0 / 2.7	4.2 / 2.8	4.6 / 3.1	4.8 / 3.2	4.6 / 3.2	4.6 / 3.2	4.6 / 3.2	4.7 / 3.3	<0.001
**VHD**													
With AF	21.6 / 24.1	24.5 / 26.5	22.8 / 25.4	22.0 / 24.6	21.1 / 22.8	19.0 / 20.0	19.7 / 21.0	18.3 / 20.2	16.6 / 18.5	15.8 / 17.3	16.6 / 17.9	16.1 / 17.7	<0.001
Without AF	0.2 / 0.1	0.2 / 0.2	0.2 / 0.2	0.3 / 0.2	0.2 / 0.2	0.3 / 0.2	0.3 / 0.2	0.3 / 0.2	0.3 / 0.2	0.3 / 0.2	0.3 / 0.2	0.3 / 0.2	<0.001
**Cardiomyopathy**													
With AF	4.4 / 3.9	5.5 / 5.0	2.8 / 2.5	3.1 / 2.9	3.2 / 3.1	2.1 / 2.1	3.0 / 2.8	2.9 / 2.9	2.6 / 2.8	3.0 / 3.1	3.1 / 3.5	3.3 / 3.6	0.047
Without AF	0.1 / 0.1	0.1 / 0.1	0.1 / 0.0	0.1 / 0.1	0.1 / 0.0	0.1 / 0.1	0.1 / 0.1	0.1 / 0.1	0.1 / 0.1	0.1 / 0.1	0.1 / 0.1	0.1 / 0.1	<0.001
**Hypertension**													
With AF	79.3 / 74.2	78.8 / 72.6	80.5 / 74.5	81.5 / 77.1	83.4 / 78.3	80.4 / 75.6	81.0 / 76.1	82.2 / 77.2	83.1 / 78.3	81.2 / 76.3	84.4 / 79.2	86.1 / 83.0	<0.001
Without AF	13.3 / 8.7	15.2 / 9.9	16.7 / 11.0	18.0 / 11.9	18.8 / 12.6	19.6 / 13.3	21.8 / 14.8	22.6 / 15.6	21.7 / 15.4	23.9 / 16.9	24.3 / 17.4	24.6 / 17.8	<0.001
**DM**													
With AF	28.0 / 25.0	33.0 / 29.9	32.9 / 29.6	34.4 / 30.4	36.8 / 33.5	37.4 / 34.7	37.2 / 34.6	39.8 / 37.2	39.0 / 36.0	36.9 / 33.4	38.8 / 36.6	40.8 / 38.4	<0.001
Without AF	6.6 / 4.7	7.6 / 5.4	8.2 / 5.8	9.3 / 6.7	8.9 / 6.3	9.1 / 6.5	10.0 / 7.1	10.5 / 7.6	10.4 / 7.7	10.7 / 7.9	11.0 / 8.2	11.5 / 8.8	<0.001

Data reported as n(%)

DM, diabetes mellitus; HF, heart failure; IHD, ischemic heart disease; VHD, valvular heart disease.

P-value obtained by Cochran-Armitage trend test

eTable 4. $CHADS_2$ and CHA_2DS_2-VASc score and oral anticoagulant therapy among AF patients during 2002–2013

Characteristics	2002 (n=1,533)	2003 (n=2,070)	2004 (n=2,409)	2005 (n=2,740)	2006 (n=2,816)	2007 (n=3,086)	2008 (n=3,361)	2009 (n=3,631)	2010 (n=3,844)	2011 (n=4,446)	2012 (n=4,822)	2013 (n=5,213)
**$CHADS_2$ score**												
0	194 (12.7)	239 (11.6)	255 (10.6)	267 (9.7)	253 (9.0)	307 (10.0)	333 (9.9)	352 (9.7)	334 (8.7)	452 (10.2)	395 (8.2)	365 (7.0)
1	387 (25.2)	501 (24.2)	598 (24.8)	710 (25.9)	720 (25.6)	741 (24.0)	804 (23.9)	799 (22.0)	818 (21.3)	1,013 (22.8)	1,097 (22.8)	1,135 (21.8)
2	450 (29.4)	598 (28.9)	618 (25.7)	698 (25.5)	744 (26.4)	793 (25.7)	823 (24.5)	929 (25.6)	1,010 (26.3)	1,108 (24.9)	1,226 (25.4)	1,328 (25.5)
3	282 (18.4)	372 (18.0)	481 (20.0)	530 (19.3)	533 (18.9)	590 (19.1)	669 (19.9)	725 (20.0)	776 (20.2)	843 (19.0)	958 (19.9)	1,034 (19.8)
4	135 (8.8)	208 (10.1)	274 (11.4)	285 (10.4)	317 (11.3)	374 (12.1)	422 (12.6)	487 (13.4)	523 (13.6)	573 (12.9)	654 (13.6)	746 (14.3)
5	68 (4.4)	127 (6.1)	147 (6.1)	204 (7.5)	200 (7.1)	218 (7.1)	240 (7.1)	251 (6.9)	297 (7.7)	347 (7.8)	363 (7.5)	452 (8.7)
6	17 (1.1)	25 (1.2)	36 (1.5)	46 (1.7)	49 (1.7)	63 (2.0)	70 (2.1)	88 (2.4)	86 (2.2)	110 (2.5)	129 (2.7)	153 (2.9)
Mean (SD)	2.03 (1.37)	2.14 (1.42)	2.21 (1.44)	2.24 (1.46)	2.26 (1.45)	2.29 (1.48)	2.31 (1.49)	2.36 (1.49)	2.41 (1.48)	2.35 (1.52)	2.41 (1.49)	2.50 (1.50)
**CHA_2DS_2-VASc score**												
0	75 (4.9)	90 (4.4)	100 (4.2)	123 (4.5)	111 (3.9)	115 (3.7)	139 (4.1)	147 (4.1)	146 (3.8)	203 (4.6)	172 (3.6)	168 (3.2)
1	220 (14.4)	283 (13.7)	327 (13.6)	367 (13.4)	377 (13.4)	410 (13.3)	423 (12.6)	412 (11.4)	417 (10.9)	531 (11.9)	554 (11.5)	559 (10.7)
2	315 (20.6)	420 (20.3)	467 (19.4)	532 (19.4)	519 (18.4)	554 (18.0)	616 (18.3)	625 (17.2)	631 (16.4)	715 (16.1)	766 (15.9)	793 (15.2)
3	331 (21.6)	444 (21.5)	450 (18.7)	511 (18.7)	547 (19.4)	557 (18.1)	596 (17.7)	672 (18.5)	709 (18.4)	820 (18.4)	886 (18.4)	963 (18.5)
4	275 (17.9)	314 (15.2)	415 (17.2)	427 (15.6)	477 (16.9)	569 (18.4)	589 (17.5)	655 (18)	696 (18.1)	775 (17.4)	872 (18.1)	944 (18.1)
5	165 (10.8)	259 (12.5)	321 (13.3)	392 (14.3)	363 (12.9)	404 (13.1)	469 (14.0)	499 (13.7)	582 (15.1)	616 (13.9)	724 (15.0)	805 (15.4)
6	90 (5.9)	152 (7.3)	196 (8.1)	208 (7.6)	254 (9.0)	270 (8.8)	304 (9.0)	360 (9.9)	383 (10.0)	456 (10.3)	494 (10.2)	516 (9.9)
7	41 (2.7)	86 (4.2)	91 (3.8)	126 (4.6)	116 (4.1)	152 (4.9)	155 (4.6)	180 (5.0)	202 (5.3)	224 (5.0)	233 (4.8)	343 (6.6)
8	19 (1.2)	20 (1.0)	36 (1.5)	45 (1.6)	44 (1.6)	49 (1.6)	63 (1.9)	67 (1.9)	69 (1.8)	97 (2.2)	107 (2.2)	113 (2.2)
9	2 (0.1)	2 (0.1)	6 (0.3)	9 (0.3)	8 (0.3)	6 (0.2)	7 (0.2)	14 (0.4)	9 (0.2)	9 (0.2)	14 (0.3)	9 (0.2)
Mean (SD)	3.11 (1.77)	3.24 (1.83)	3.33 (1.87)	3.36 (1.92)	3.39 (1.89)	3.44 (1.90)	3.46 (1.92)	3.55 (1.93)	3.60 (1.91)	3.55 (1.96)	3.61 (1.92)	3.71 (1.93)
**OAC therapy**	421 (27.5)	606 (29.3)	749 (31.1)	882 (32.2)	955 (33.9)	1017 (33.0)	1133 (33.7)	1288 (35.5)	1331 (34.6)	1548 (34.8)	1705 (35.4)	1929 (37.0)

Data reported as n(%)
OAC, oral anticoagulant therapy; SD, standard deviation.

eTable 5. Age-specific prevalence of hypertension, DM and IHD by sex and age group among AF patients during 2002-2013

Comorbidities	Year												***P* for trend**	**β (*P*-value)***
	**2002**	**2003**	**2004**	**2005**	**2006**	**2007**	**2008**	**2009**	**2010**	**2011**	**2012**	**2013**		
**Hypertension**														
Male														
30–39 years	14 (45.2)	23 (51.1)	26 (49.1)	42 (59.2)	30 (49.2)	31 (50.0)	37 (64.9)	25 (52.1)	16 (39.0)	21 (42.0)	26 (46.4)	33 (53.2)	0.267	0.22 (0.767)
40–49 years	60 (66.7)	89 (67.4)	99 (63.9)	108 (67.5)	106 (68.4)	97 (64.7)	112 (63.3)	111 (65.3)	95 (57.9)	94 (50.5)	131 (63.9)	132 (68.0)	0.043	3.85 (0.008)
50–59 years	129 (76.3)	161 (74.9)	200 (79.1)	233 (76.4)	248 (78.7)	258 (79.9)	251 (72.3)	269 (73.5)	297 (77.5)	357 (73.6)	403 (78.6)	436 (77.4)	0.468	24.58 (<0.001)
60–69 years	225 (76.8)	310 (80.3)	345 (78.6)	399 (82.8)	412 (83.6)	412 (80.3)	469 (80.9)	499 (81.3)	533 (81.9)	555 (81.0)	559 (82.1)	637 (84.7)	0.008	32.28 (<0.001)
70–79 years	134 (79.3)	187 (80.3)	238 (81.8)	319 (87.6)	343 (85.1)	413 (82.8)	458 (82.8)	495 (84.8)	536 (83.2)	635 (81.6)	784 (86.2)	886 (88.1)	0.002	62.97 (<0.001)
≥80 years	37 (74.0)	62 (81.6)	83 (87.4)	93 (80.9)	103 (83.1)	131 (83.4)	172 (85.1)	187 (86.2)	212 (85.8)	247 (86.7)	292 (90.7)	326 (89.1)	<0.001	25.47 (<0.001)
Female														
30–39 years	11 (45.8)	9 (31.0)	13 (37.1)	17 (48.6)	12 (46.2)	10 (31.2)	11 (36.7)	9 (27.3)	8 (25.8)	6 (22.2)	5 (19.2)	7 (29.2)	0.005	−0.64 (0.012)
40–49 years	42 (61.8)	42 (53.8)	38 (52.8)	56 (65.1)	39 (54.9)	43 (60.6)	49 (59.8)	46 (53.5)	45 (60.0)	44 (57.1)	37 (48.7)	51 (69.9)	0.401	0.24 (0.633)
50–59 years	88 (70.4)	120 (71.4)	113 (67.3)	127 (72.2)	148 (74.4)	134 (70.9)	118 (69.4)	158 (76.7)	161 (70.0)	157 (67.7)	168 (69.1)	196 (76.9)	0.269	7.39 (<0.001)
60–69 years	205 (83.3)	259 (82.2)	307 (87.0)	288 (83.5)	294 (85.5)	315 (81.8)	341 (81.0)	343 (81.7)	359 (84.3)	342 (77.9)	372 (82.7)	383 (80.5)	0.013	13.11 (<0.001)
70–79 years	163 (87.2)	236 (89.4)	289 (88.1)	363 (87.9)	368 (90.2)	396 (85.9)	414 (86.2)	494 (87.6)	532 (89.1)	629 (86.5)	718 (90.2)	762 (91.6)	0.028	50.87 (<0.001)
≥80 years	71 (87.7)	109 (84.5)	144 (86.2)	162 (86.2)	194 (89.4)	213 (87.3)	238 (90.8)	291 (90.1)	319 (89.9)	427 (89.9)	503 (92.3)	556 (91.1)	<0.001	41.83 (<0.001)
**DM**														
Male														
30–39 years	7 (22.6)	5 (11.1)	5 (9.4)	14 (19.7)	12 (19.7)	8 (12.9)	11 (19.3)	6 (12.5)	6 (14.6)	12 (24.0)	9 (16.1)	8 (12.9)	0.470	0.143 (0.596)
40–49 years	19 (21.1)	37 (28.0)	36 (23.2)	43 (26.9)	43 (27.7)	36 (24.0)	46 (26.0)	50 (29.4)	51 (31.1)	49 (26.3)	50 (24.4)	52 (26.8)	0.258	2.25 (<0.001)
50–59 years	45 (26.6)	66 (30.7)	85 (33.6)	91 (29.8)	92 (29.2)	117 (36.2)	121 (34.9)	121 (33.1)	139 (36.3)	167 (34.4)	169 (32.9)	188 (33.4)	0.038	11.91 (<0.001)
60–69 years	85 (29.0)	137 (35.5)	157 (35.8)	174 (36.1)	167 (33.9)	193 (37.6)	221 (38.1)	252 (41.0)	264 (40.6)	275 (40.1)	272 (39.9)	302 (40.2)	<0.001	18.05 (<0.001)
70–79 years	50 (29.6)	74 (31.8)	111 (38.1)	155 (42.6)	148 (36.7)	216 (43.3)	232 (42.0)	253 (43.3)	282 (43.8)	326 (41.9)	393 (43.2)	446 (44.3)	<0.001	33.91 (<0.001)
≥80 years	13 (26.0)	18 (23.7)	33 (34.7)	46 (40.0)	41 (33.1)	58 (36.9)	83 (41.1)	84 (38.7)	107 (43.3)	113 (39.6)	131 (40.7)	163 (44.5)	<0.001	12.89 (<0.001)
Female														
30–39 years	0 (0.0)	4 (13.8)	5 (14.3)	2 (5.7)	3 (11.5)	6 (18.8)	5 (16.7)	2 (6.1)	5 (16.1)	1 (3.7)	5 (19.2)	3 (12.5)	0.192	0.163 (0.603)
40–49 years	12 (17.6)	12 (15.4)	5 (6.9)	15 (17.4)	15 (21.1)	15 (21.1)	18 (22.0)	26 (30.2)	18 (24.0)	19 (24.7)	19 (25.0)	21 (28.8)	<0.001	1.09 (0.006)
50–59 years	30 (24.0)	56 (33.3)	52 (31.0)	48 (27.3)	53 (26.6)	53 (28.0)	47 (27.6)	62 (30.1)	60 (26.1)	52 (22.4)	70 (28.8)	74 (29.0)	0.261	2.42 (0.004)
60–69 years	78 (31.7)	104 (33.0)	127 (36.0)	124 (35.9)	136 (39.5)	156 (40.5)	164 (39.0)	174 (41.4)	178 (41.8)	155 (35.3)	170 (37.8)	181 (38.0)	0.030	8.09 (<0.001)
70–79 years	62 (33.2)	110 (41.7)	134 (40.9)	171 (41.4)	179 (43.9)	199 (43.2)	212 (44.2)	259 (45.9)	261 (43.7)	305 (42.0)	353 (44.3)	390 (46.9)	0.003	26.91 (<0.001)
≥80 years	23 (28.4)	38 (29.5)	47 (28.1)	67 (35.6)	79 (36.4)	88 (36.1)	91 (34.7)	127 (39.3)	147 (41.4)	198 (41.7)	212 (38.9)	258 (42.3)	<0.001	20.12 (<0.001)
**IHD**														
Male														
30–39 years	11 (35.5)	11 (24.4)	14 (26.4)	22 (31.0)	16 (26.2)	18 (29.0)	18 (31.6)	14 (29.2)	7 (17.1)	13 (26.0)	10 (17.9)	18 (29.0)	0.140	−0.07 (0.855)
40–49 years	40 (44.4)	53 (40.2)	59 (38.1)	66 (41.2)	64 (41.3)	53 (35.3)	64 (36.2)	63 (37.1)	63 (38.4)	61 (32.8)	66 (32.2)	55 (28.4)	<0.001	1.01 (0.114)
50–59 years	73 (43.2)	94 (43.7)	124 (49.0)	125 (41.0)	143 (45.4)	137 (42.4)	129 (37.2)	143 (39.1)	158 (41.3)	161 (33.2)	182 (35.5)	175 (31.1)	<0.001	8.15 (<0.001)

60–69 years	149 (50.9)	182 (47.2)	189 (43.1)	223 (46.3)	219 (44.4)	228 (44.4)	245 (42.2)	267 (43.5)	270 (41.5)	257 (37.5)	249 (36.6)	278 (37.0)	<0.001	10.12 (<0.001)
70–79 years	82 (48.5)	92 (39.5)	139 (47.8)	186 (51.1)	174 (43.2)	249 (49.9)	236 (42.7)	269 (46.1)	314 (48.8)	348 (44.7)	396 (43.6)	434 (43.1)	0.052	31.41 (<0.001)
≥80 years	19 (38.0)	37 (48.7)	48 (50.5)	56 (48.7)	60 (48.4)	82 (52.2)	100 (49.5)	109 (50.2)	118 (47.8)	114 (40.0)	147 (45.7)	144 (39.3)	0.006	11.55 (<0.001)
Female														
30–39 years	4 (16.7)	3 (10.3)	9 (25.7)	8 (22.9)	2 (7.7)	5 (15.6)	4 (13.3)	5 (15.2)	6 (19.4)	4 (14.8)	2 (7.7)	2 (8.3)	0.110	−0.24 (0.228)
40–49 years	22 (32.4)	17 (21.8)	13 (18.1)	21 (24.4)	18 (25.4)	22 (31.0)	22 (26.8)	21 (24.4)	19 (25.3)	22 (28.6)	15 (19.7)	19 (26.0)	0.467	0.038 (0.887)
50–59 years	58 (46.4)	72 (42.9)	59 (35.1)	68 (38.6)	69 (34.7)	63 (33.3)	47 (27.6)	74 (35.9)	65 (28.3)	65 (28.0)	57 (23.5)	75 (29.4)	<0.001	0.273 (0.708)
60–69 years	125 (50.8)	145 (46.0)	158 (44.8)	151 (43.8)	152 (44.2)	162 (42.1)	195 (46.3)	173 (41.2)	187 (43.9)	163 (37.1)	175 (38.9)	160 (33.6)	<0.001	3.38 (0.023)
70–79 years	99 (52.9)	142 (53.8)	160 (48.8)	206 (49.9)	195 (47.8)	218 (47.3)	206 (42.9)	258 (45.7)	261 (43.7)	291 (40.0)	338 (42.5)	338 (40.6)	<0.001	20.15 (<0.001)
≥80 years	38 (46.9)	74 (57.4)	90 (53.9)	92 (48.9)	98 (45.2)	128 (52.5)	108 (41.2)	146 (45.2)	165 (46.5)	202 (42.5)	220 (40.4)	255 (41.8)	<0.001	17.39 (<0.001)

Data reported as n(%)

AF, atrial fibrillation; DM, diabetes mellitus; IHD, ischemic heart disease.

P for trend obtained by Cochran-Armitage trend test; β (*P*-value) obtained by linear regression analysis.
